# Demographic Characteristics and County-level Indicators of Social Vulnerability in Salmonellosis Outbreaks Linked to Ground Beef—United States, 2012–2018

**DOI:** 10.1016/j.jfp.2024.100411

**Published:** 2024-11-20

**Authors:** Michelle A. Waltenburg, Zainab Salah, Michelle Canning, Kelly McCain, David Rickless, Michael Ablan, Tamara N. Crawford, Mabel Sheau Fong Low, Misha Robyn, Noelle Angelique M. Molinari, Katherine E. Marshall

**Affiliations:** 1 Centers for Disease Control and Prevention, 1600 Clifton Rd NE, Atlanta, GA 30329, USA; 2 Oak Ridge Institute for Science and Education, 1299 Bethel Valley Rd, Oak Ridge, TN 37830, USA

**Keywords:** Foodborne illness, Salmonella, Social vulnerability

## Abstract

Ground beef is a common source of US *Salmonella* illnesses and outbreaks. However, the demographic and socioeconomic factors that are related to risk in ground beef-associated outbreaks of *Salmonella* infections are poorly understood. We describe the individual-level demographic characteristics and county-level indicators of social vulnerability for people infected with *Salmonella* linked to outbreaks associated with ground beef in the United States during 2012–2018. Non-Hispanic (NH) White and NH American Indian/Alaska Native persons, and people living in nonmetropolitan areas, were overrepresented among people in salmonellosis outbreaks linked to ground beef. Case patients disproportionately resided in counties with high social vulnerability, suggesting that one or more community social risk factors may contribute to or be associated with some food safety risks. Collecting and analyzing socioeconomic and demographic characteristics of people in outbreaks can help identify disparities in foodborne disease, which can be further characterized and inform equity-focused interventions.

Nontyphoidal *Salmonella* cause an estimated 1.4 million illnesses, 26,000 hospitalizations, and 400 deaths annually in the United States (Collier et al., 2021). *Salmonella* infection can occur through exposure to contaminated food, water, environment, or infected animals. Salmonellosis outbreaks linked to ground beef were first identified in 1975; in 2021, approximately 6% of all *Salmonella*-related foodborne illnesses were attributed to ground beef (Laufer et al., 2015; IFSAC, 2023).

*Salmonella* is not currently classified as an adulterant in meat and poultry that is not ready-to-eat; therefore, its presence in ground beef is permissible (USDA, 2015). Food safety education interventions and tailored health messaging that incorporate behavior change theories (e.g., Theory of Planned Behavior (Young et al., 2017)) and focus on safe handling and preparation of raw meat and poultry could help reduce the burden of foodborne illness (Redmond & Griffith, 2003). However, education and health messaging alone will not be effective unless they incorporate social and cultural factors that influence access to safe foods, ability to safely store and prepare foods, and other food safety behaviors (Fein et al., 1988).

Many factors, including where people live, their environment, their housing, income, education, and work can all influence health (i.e., determinants of health). Health disparities occur when differences in health outcomes are related to social or demographic factors. The social factors (e.g., demographic and socioeconomic factors such as poverty, lack of access to transportation, crowded housing, etc.) that influence risk in ground beef-associated *Salmonella* outbreaks are poorly understood. Collecting and analyzing data on demographic and socioeconomic characteristics such as household income, education, and language spoken among people linked to foodborne illness outbreaks can potentially identify which factors might be associated with health disparities (Quinlan, 2023; Patil et al., 2005; Hadler et al., 2020; Whitney et al., 2015). However, these data have rarely been collected during routine foodborne illness outbreak investigations.

The Centers for Disease Control and Prevention/Agency for Toxic Substances and Disease Registry Social Vulnerability Index (CDC/ATSDR SVI) uses data on demographic and socioeconomic factors to identify US counties that are especially vulnerable to the economic and social consequences of a public health emergency (Flanagan et al., 2018). More recently, CDC/ATSDR SVI has been used to better understand social and health disparities in relation to the COVID-19 pandemic (Islam et al., 2021; Barry et al., 2021). Its value in identifying areas at particular risk for foodborne illness and disease outbreaks warrants exploration.

This report describes the individual-level demographic characteristics of people in salmonellosis outbreaks linked to ground beef in the United States during 2012–2018 and uses county-level indicators of social vulnerability to assess community characteristics and determine if patients reside in counties that are systematically more vulnerable to economic and social consequences of emergencies, including disease outbreaks, than the average US county. We aim to identify possible disparities in ground beef–associated *Salmonella* outbreaks and identify whether demographic or socioeconomic characteristics might be related to increased risk to inform more focused consumer-based and other public health interventions that incorporate social and cultural factors.

## Materials and methods

### Data sources.

We identified *Salmonella* outbreaks linked to ground beef using CDC’s Foodborne Disease Outbreak Surveillance System (FDOSS). We included outbreaks that met the following inclusion criteria: laboratory-confirmed etiology of *Salmonella*, ground beef listed as the single contaminated ingredient or implicated food, and first reported illness onset occurred January 1, 2012–December 31, 2018. We defined a case as illness in a person linked to an outbreak as determined by jurisdictional investigators; we defined outbreaks using CDC FDOSS definitions (i.e., two or more illnesses resulting from the consumption of a common food) and designated them as single or multistate (CDC, 2023).

We obtained additional information about each outbreak (e.g., type of ground beef implicated, how ground beef was prepared) from FDOSS. We obtained patient demographic information (age, sex, race, and ethnicity) for laboratory-confirmed cases through the System for Enteric Disease Response, Investigation, and Coordination (SEDRIC) (CDC, 2024a). We obtained data on patient county of residence from PulseNet, the national molecular subtyping network for foodborne disease surveillance in the United States (CDC, 2024b). However, information on patient race and ethnicity in SEDRIC during this period was largely missing, and the county listed in PulseNet may have corresponded to where the specimen was collected or where testing occurred, rather than of patient residence. Therefore, CDC requested that state health departments provide missing data and verify patient race, ethnicity, and county of residence. This information was received from 94% (44/47) of states.

We combined race and ethnicity into a single variable. Data were collected in a combined Asian or Pacific Islander (API) category and could not be separated for analysis. We first classified patients of any race with Hispanic or Latino ethnicity as “Hispanic” (any race). We classified patients with non-Hispanic (NH) ethnicity as NH American Indian or Alaska Native (AI/AN), NH API, NH Black, or NH White. Patients who identified with NH ethnicity and either multiple races or another race were classified as NH multiple or another race(s). Patients who did not report ethnicity but reported race, patients who did not report race but reported ethnicity, and patients who did not report neither race nor ethnicity were categorized as Unknown for the purposes of this analysis.

We classified patient county of residence by rurality using the 2013 National Center for Health Statistics (NCHS) Urban-Rural Classification Scheme for Counties (Ingram & Franco, 2014). We aggregated large central metropolitan/city, large fringe metropolitan/suburb, medium metropolitan, and small metropolitan county categories into a single metropolitan category; we aggregated micropolitan and noncore/rural county categories into a single nonmetropolitan category.

**Figure 1.**
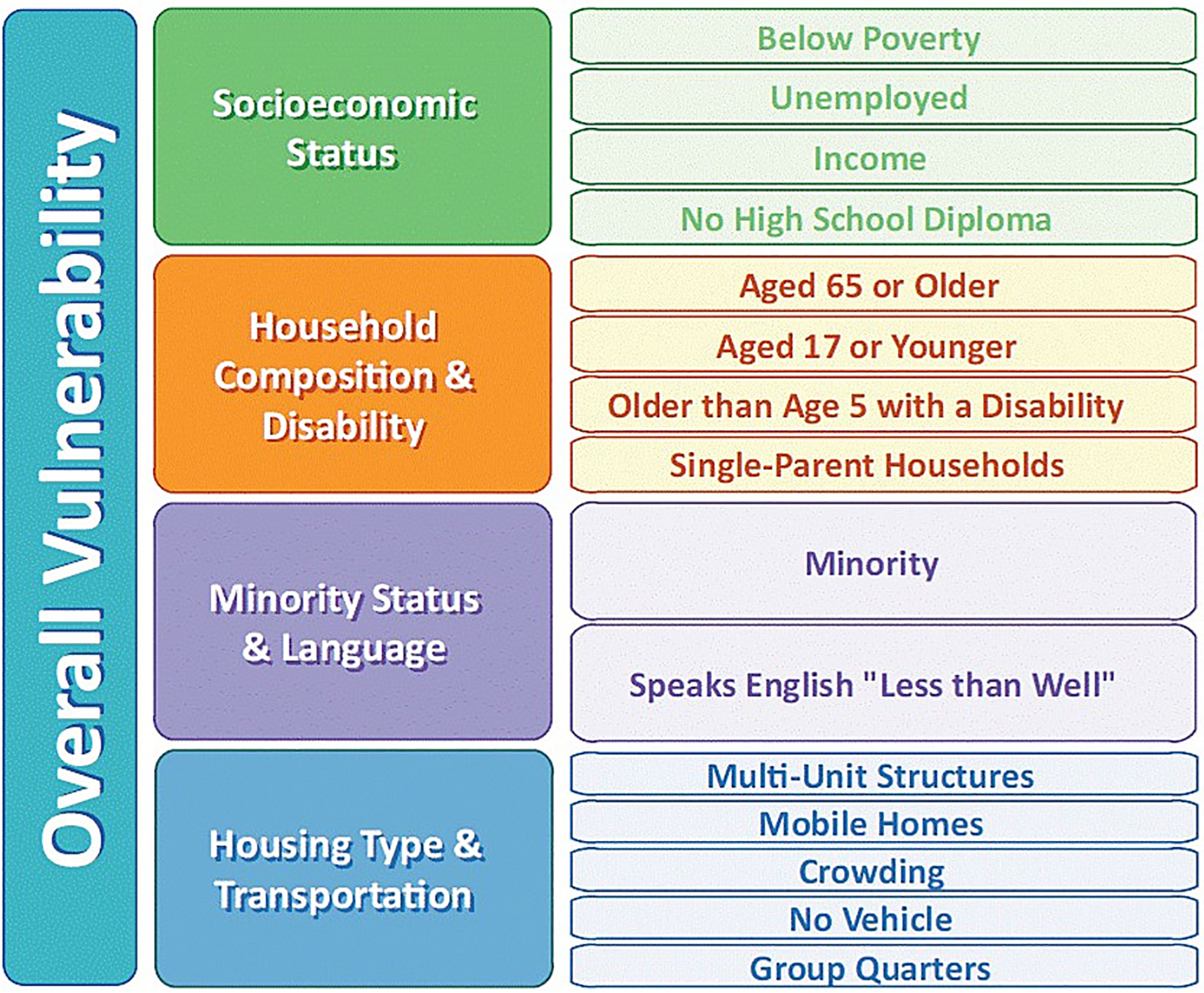
Centers for Disease Control and Prevention/Agency for Toxic Substances and Disease Registry Social Vulnerability Index themes and variables. The CDC/ATSDR SVI ranks each county on 15 population-based social determinants of health measures and groups them into four themes: Theme 1: Socioeconomic Status, Theme 2: Household Composition and Disability, Theme 3: Minority Status and Language, and Theme 4: Housing Type and Transportation.

Sociodemographic information, such as household income and size, housing type, transportation, and other information is not typically collected during outbreak investigations. We used the CDC/ATSDR SVI as an area-based proxy measure for patient sociodemographic characteristics, where social vulnerability is defined as the demographic and socioeconomic factors that adversely affect communities that encounter stressors, including disease outbreaks (CDC, 2024c). The CDC/ATSDR SVI ranks each county on 15 population-based measures and groups them into four themes: Theme 1: Socioeconomic Status, Theme 2: Household Composition and Disability, Theme 3: Minority Status and Language, and Theme 4: Housing Type and Transportation (Fig. 1). The index includes a percentile rank from 0 to 1 for each theme, as well as an overall percentile rank. Higher ranks indicate higher social vulnerability. For example, a ranking of 0.7 corresponds with the 70th percentile, indicating a county with greater vulnerability than 70 percent of US counties. The original CDC/ATSDR SVI was created in 2010; data used to calculate the SVI were updated in 2014, 2016, and 2018, based on US Census Bureau and American Community Survey data releases. We determined the CDC/ATSDR SVI county rank using the most recent iteration that corresponded to the patient’s year of illness onset.

### Statistical methods.

We examined outbreak characteristics (single state vs. multistate, number of laboratory-confirmed illnesses, hospitalizations, and deaths) and calculated frequencies for patient characteristics (age, sex, race/ethnicity, geographic classification) by outbreak. When age, sex, race/ethnicity, or patient residence were unknown, the data were considered missing and excluded from respective analyses. We calculated proportional distributions for patients and the total US population by race/ethnicity, geographic classification, and overall county-level CDC/ATSDR SVI ranking. We obtained proportional distributions for the total US population using the 2015–2019 American Community Survey (ACS) 5-year estimates (United States Census Bureau, 2021). We compared proportional distributions of race/ethnicity and geographic classification among patients to the total US population using Pearson’s chi square tests or Fisher’s exact tests when necessary.

We weighted county-level SVI scores for counties with multiple patients by assigning each individual patient an SVI score that corresponded with their county of residence, from the most recent iteration that matched their illness onset date. As 56% of patients were associated with one outbreak (Outbreak 10, Table 1), a sensitivity analysis was performed excluding this outbreak to assess its impact on these results. We calculated the mean and 95% confidence intervals (CIs) of the overall CDC/ATSDR SVI rank and each theme. Using a one-sample inferiority *t*-test, we compared the weighted mean SVI county-level rank among patients to the mean SVI rank of all US counties (0.5; moderate vulnerability). We report results for the analysis of all patients but note when the sensitivity analysis identified different findings. All analyses were performed using SAS version 9.4 (https://www.sas.com).

We mapped overall CDC/ATSDR SVI, SVI theme ranks, and case rates by quartile among all counties where ground beef outbreak-associated patients resided using R version 3.6.3 (R Foundation for Statistical Computing, https://www.r-project.org) and ArcGIS Pro version 2.6.4 (Esri Inc.).

### Ethics.

This activity was reviewed by the US Centers for Disease Control and Prevention and conducted in accordance with applicable federal law and CDC policy (HHS, 2021).

## Results

### Outbreaks.

Overall, 10 *Salmonella* outbreaks were linked to ground beef during 2012–2018, resulting in 737 illnesses, 206 hospitalizations, and one death. The median outbreak size was 32 illnesses (range 3–436). Outbreak 10 accounted for 56% (436/737) of all illnesses. Four outbreaks were single state and six outbreaks involved multiple states. The median number of states involved in multistate outbreaks was 16 (range: 6–30).

Three outbreaks were associated with ground beef dishes traditionally served raw. Outbreak 2 was associated with kibbeh, a dish of Middle Eastern origin which is sometimes served raw (Table 1). Outbreak 8 was associated with kitfo, a traditional Ethiopian dish which contains minced raw beef. Outbreak 9 was associated with a traditional Christmas dish popular in parts of the Upper Midwest, thought to be of Northern European origin, known as “Cannibal sandwiches” (CDC, 1995); all patients consumed raw ground beef served on bread with raw onion.

### Individual-level demographic characteristics.

**Figure 2.**
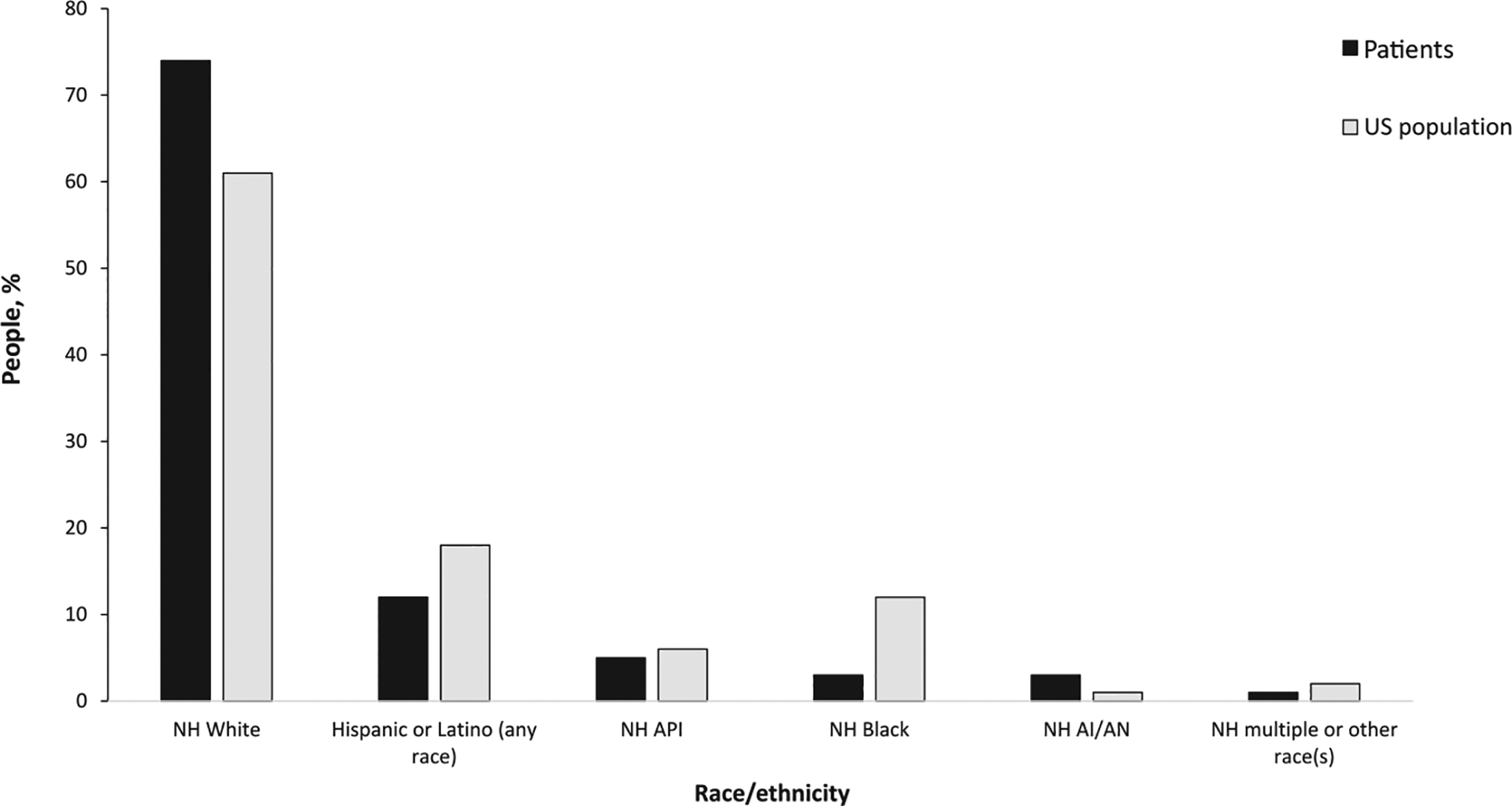

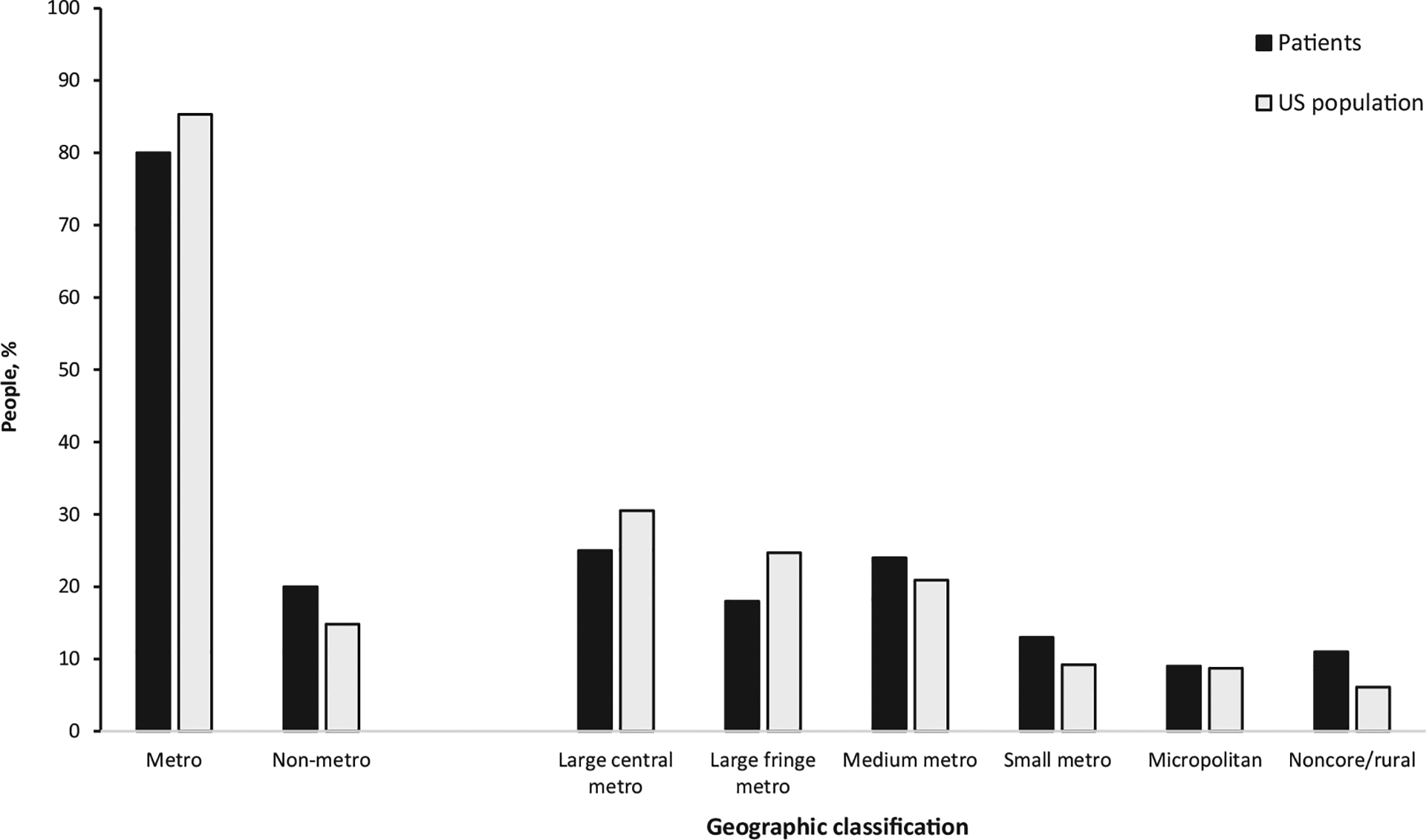
Percentage of people infected with *Salmonella* linked to ground beef outbreaks in the United States from 2012 to 2018 and US population by race/ethnicity and geographic classification. Proportional distributions were calculated after excluding missing and unknown values; race/ethnicity information was available for 79% (579/737) of patients and location information was available for 98% (720/737) of patients. Proportional distributions for the overall US population were calculated using the 2014–2018 American Community Survey (United States Census Bureau, 2021). Patient county of residence was merged with the 2013 National Center for Health Statistics Urban-Rural Classification Scheme for Counties (Ingram & Franco, 2014). Patients who resided in large central metropolitan/city, large fringe metropolitan/suburb, medium metropolitan, and small metropolitan counties were considered metropolitan; patients who resided in micropolitan and noncore/rural counties were considered nonmetropolitan. NH, non-Hispanic; API, Asian or Pacific Islander; AI/AN, American Indian or Alaska Native; metro, metropolitan.

Among patients with available demographic information, 50% (365/730) were female (Table 1). Across the outbreaks, the percent of female patients ranged from 0 to 100% (IQR: 49–59). Of 730 patients with information on age, median age across all outbreaks was 41 years (range: <1–101); 52 (7%) patients were <5 years and 152 (21%) were ≥65 years. Race and ethnicity information were available for 87% (640/737) and 79% (582/737) of patients, respectively; combined race/ethnicity information was available for 79% (579/737) of patients. Among these patients, 429 (74%) were NH White, 72 (12%) were Hispanic, 30 (5%) were NH API, 20 (3%) were NH Black, 20 (3%) were NH AI/AN, and 8 (1%) were NH multiple or another race(s). Compared with the total US population, a higher proportion of patients were NH White (74% vs. 61%; *p* < 0.001) or NH AI/AN (3% vs. 1%; *p* < 0.001) (Fig. 2). In four outbreaks, all patients with known race/ethnicity reported the same race/ethnicity (Outbreak 3, NH API; Outbreaks 5 and 9, NH White; and outbreak 8, NH Black). County of residence was available for 98% (720/737) of patients; these patients represented 278 counties (median: 1 patient per county; range 1–37). Among the 720 patients, 179 (25%) lived in large central metropolitan counties, 131 (18%) lived in large fringe metropolitan counties, 170 (24%) lived in medium metropolitan counties, 96 (13%) lived in small metropolitan counties, 63 (9%) lived in micropolitan counties, and 81 (11%) lived in noncore/rural counties (Table 1). Overall, 576 (80%) lived in metropolitan counties and 144 (20%) lived in nonmetropolitan counties. Compared with the total US population, a higher proportion of patients resided in nonmetropolitan counties (20% vs. 15%; *p* < 0.001) (Fig. 2).

### County-level indicators of social vulnerability.

**Figure 3.**
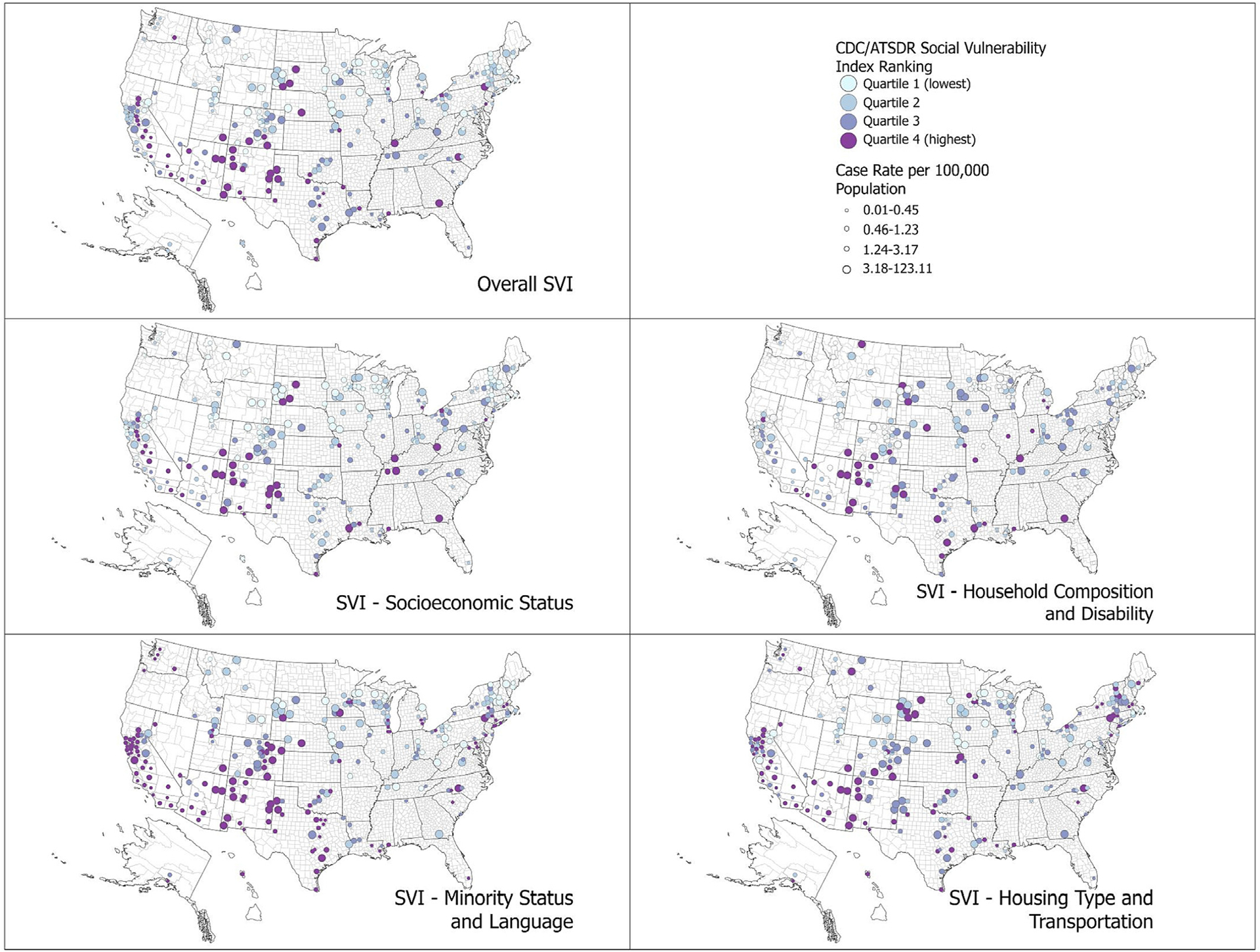
County social vulnerability index and case rate quartiles of people infected with *Salmonella* linked to ground beef outbreaks (*n* = 720 patients; *n* = 278 counties)—United States, 2012–2018. The CDC/ATSDR SVI ranks each county on 15 population-based social determinants of health measures and groups them into four themes: Theme 1: Socioeconomic Status, Theme 2: Household Composition and Disability, Theme 3: Minority Status and Language, and Theme 4: Housing Type and Transportation. Scores for CDC/ATSDR SVI measures (overall and for each theme) represent percentile ranks by county from 0 to 1; higher scores indicate higher vulnerability; percentile ranks were analyzed as quartiles. The analytic dataset excluded patients who were missing information on county of residence (*n* = 17). County population within the SVI dataset was used to calculate ground beef outbreak case rates per 100,000 population. Case rates were analyzed as quartiles: first quartile (0.19–0.45 per 100,000; lowest), second quartile (0.46–1.23 per 100,000), third quartile (1.24–3.17 per 100,000), and fourth quartile (above 3.18 per 100,000; highest). SVI, social vulnerability index.

The proportion of patients residing in counties in each SVI quartile varied by outbreak (Table 2). Across all outbreaks for overall SVI, the percent of patients residing in counties with the highest overall SVI ranking (fourth quartile) ranged from 0 to 42% (interquartile range [IQR]: 2–20). The weighted mean overall SVI rank of patient counties was higher than the mean among all US counties, though this difference was small (0.53 vs. 0.50; *p* < 0.001) (Table 3) and was no longer significant when a sensitivity analysis was performed removing the largest outbreak (outbreak 10, Appendix Table 3). The weighted mean county-level SVI rank was higher among patient counties than among all US counties for SVI Theme 3 Minority Status and Language (0.76 vs. 0.50; *p* < 0.001) and Theme 4 Housing Type and Transportation (0.64 vs. 0.50; *p* < 0.001) (Table 3). No significant difference was found for SVI Theme 1 Socioeconomic Status or Theme 2 Household Composition and Disability. The distribution of cases by county and SVI quartile is shown in Fig. 3.

## Discussion

Collecting and analyzing individual-level demographic characteristics and county-level indicators of social vulnerability among people with foodborne illnesses can identify factors associated with health disparities and ultimately enable more focused public health interventions. NH White people, NH AI/AN people, and people living in nonmetro counties were overrepresented among people in salmonellosis outbreaks linked to ground beef. Patients lived in counties that had higher vulnerability rankings for minority status and language and housing type and transportation than the mean of all US counties, suggesting that some aspects of community social vulnerability may contribute to some ground beef food safety risks. This report demonstrates how the social vulnerability framework might be useful to better understand who is affected by foodborne illness outbreaks in the United States.

NH White persons were overrepresented among people linked to ground beef-associated outbreaks. This finding could be partly due to differences in consumption of ground beef, and differences in opportunities for exposure to contaminated ground beef. Data from a survey of healthy people conducted in 15 US sites during 2018–2019 showed that NH White persons were the most likely to report eating ground beef in the previous week (71.5%), followed by Hispanic persons (63.7%), NH Black persons (58.4%), and people of another race (53.4%); data for AI/AN persons were not provided (Centers for Disease Control and Prevention (2022)). Differences in preferences for or cultural traditions around eating raw or undercooked ground beef could also contribute to our findings. From a meta-analysis of 20 studies, White persons (22%) were most likely to report consuming raw or undercooked ground beef compared with people who are Hispanic (14%), Asian (14%), or Black (7%); ground beef consumption information was not available for AI/AN people (Patil et al., 2005).

People with salmonellosis linked to ground beef lived in counties that had higher vulnerability rankings for the minority status and language (speaking English “less than well”) theme. This might be due to limited availability of food safety information for handling and storage of ground beef and training for those who store and handle ground beef in restaurants and grocery stores in languages other than English. Additionally, differences in preferences or cultural practices around handling and preparation of ground beef could help explain our findings if affected counties have a higher proportion of people who consume dishes traditionally made with raw beef. At least two outbreaks in this analysis were associated with culturally important raw ground beef dishes (i.e., kibbeh, kitfo). However, a separate analysis compared SVI themes for counties with people who had salmonellosis linked to ground beef with those who recently ate ground beef but did not report getting sick (Salah et al., 2024). The vulnerability rankings for the minority status and language SVI theme in that analysis were similarly high in both groups. This suggests that the observed findings may be partially attributed to increased consumption of ground beef in counties with high vulnerability related to minority status.

We found that a higher proportion of patients resided in nonmetro areas compared with the total US population. This might be partly explained by increased consumption of ground beef products in nonmetro areas. Previous studies have demonstrated that people living in rural communities consume ground beef more frequently than people living in urban communities (Taylor et al., 2012; Davis & Lin, 2005). Further, people living in rural areas may need to travel longer distances to grocery stores, which could expose ground beef to warmer temperatures, and for longer than the recommended limit (CDC, 2024d).

People linked to ground beef-associated *Salmonella* outbreaks lived in counties that were more vulnerable with respect to SVI Theme 4: Housing Type and Transportation, which includes measures for households with no vehicle available, living in mobile homes, living in a group home, or crowding (i.e., more people than rooms in a household). In the analysis where people with salmonellosis linked to ground beef were compared with people who recently ate ground beef, this SVI theme was no longer significant (Salah et al., 2024), therefore, it is possible that our findings from this analysis could be related to differences in consumption of ground beef. However, not having a vehicle and having to rely on public transportation to get to the grocery store might increase transit time and could expose ground beef to warmer temperatures for longer than the recommended limit (CDC, 2024d), allowing for growth of any pathogens that might already be present. Differences in availability, quality, and effectiveness of appliances and tools for storing and cooking ground beef related to housing type could also play a role in these findings. Refrigerators that are overcrowded or do not maintain an appropriate temperature could contribute to the growth of pathogens that are already present in ground beef. A lack of access to cooktops or ovens could contribute to challenges around cooking ground beef to 160F. Results of a survey of consumers regarding appliance use for preparing frozen stuffed chicken products showed that people who lived in mobile homes or with lower income reported lower use of ovens compared with microwaves, suggesting that economic and other factors might influence access to cooking appliances (Marshall et al., 2022). Finally, there could be differences in the availability of food safety information and training for those preparing food in group homes, which include college residence halls, residential treatment centers, nursing facilities, military barracks, correctional facilities, worker homes, and other types of group homes. A previous analysis found that people in correctional facilities face a disproportionate risk of foodborne illness outbreaks (Marlow et al., 2017). One study found that staff in group homes for people with developmental disabilities in Massachusetts lacked knowledge of safe food preparation in some key areas, including storage and handling, and identified a need for food safety training (Walter et al., 1997). While previous studies that have analyzed the associations between household-level characteristics and foodborne illness typically focus on income and education (Quinlan, 2023; Chang et al., 2009; Newman et al., 2015), our analysis suggests that housing type and transportation could also be important to consider when collecting and examining foodborne disease data and identifying interventions.

While we did not examine it in this analysis, it is possible there are differences in microbial quality of ground beef (e.g., prevalence of *Salmonella*) that retailers and consumers buy, which could influence the risk of illness. To our knowledge, no studies have examined whether there are systematic differences in the microbial quality of ground beef by retail location type (e.g., supermarket vs. small corner store), geography (i.e., region, or urban vs. rural areas), or characteristics of the communities they serve (e.g., race/ethnicity, socioeconomic status). A study conducted in the Philadelphia area did not find a difference in pathogen levels for meat and poultry across different retail types but did identify differences in microbial quality in produce at the retail level by community-level socioeconomic status (Koro et al., 2010). More research is needed to determine whether these and other differences are observed across the United States.

Given the multitude of factors, including social and structural factors, along the farm-to-fork continuum that may influence the risk for *Salmonella* infection associated with ground beef, exploring interventions that are applied to minimize contamination of ground beef in the first place and do not require modification of consumer behaviors are essential. These can include preharvest interventions such as vaccination of cattle and biosecurity, and interventions at slaughter and processing, including lymph node removal and irradiation (Edrington et al., 2020; Stuttgen, 2017; USDA, 2017; Castell-Perez and Moreira, 2020). To improve the effectiveness and acceptability of consumer-focused food safety interventions, cultural responsiveness and consideration of traditions are essential. The effectiveness of public food safety education efforts may be increased by incorporating formative research with disproportionately affected populations to understand knowledge, attitudes, perceived risks, and preferences and tailor educational strategies that include behavioral components (Young et al., 2017; Sivaramalingam et al., 2015; Siebert et al., 2014). For example, focus groups among American Indian participants identified the importance of framing education efforts around positive health rather than sickness, because associating sickness with food is culturally inappropriate (Siebert et al., 2014). Tailored health messaging and interventions have been shown to be more effective at driving long-term behavior change when compared to general, one size fits all health messaging and intervention strategies (Noar et al., 2007). The format, mechanisms, messengers, and languages for disseminating education and health messaging should also be tailored. Further, strategies that offer safer alternatives to prepare and consume culturally important or traditional foods (e.g., using irradiated ground beef for raw dishes) could be developed together with disproportionately impacted populations.

This report is subject to at least five limitations. First, analysis of individual-level demographic characteristics was performed on a small, nonrandom sample of all laboratory-confirmed salmonellosis cases that were reported as part of a ground beef-associated outbreak in CDC FDOSS. Further, state reporting to FDOSS is voluntary and it is possible that not all ground beef-associated *Salmonella* outbreaks were captured. Our findings do not represent all people who had ground beef-associated *Salmonella* infections. Second, CDC/ATSDR SVI is an area-based measure; individual patient sociodemographic characteristics may differ from the proxy measures applied to them in this analysis. Third, one outbreak (Outbreak 10) represented over half (59%) of all cases in this analysis. To assess whether observed differences in SVI themes were overly influenced by this single outbreak, we performed a sensitivity analysis excluding Outbreak 10 (Appendix). Demographic and geographic comparison interpretations were similar after excluding Outbreak 10. When assessing the weighted mean overall SVI rank of patient counties compared with the mean among all US counties, the difference for overall SVI rank was no longer significant, but the difference for SVI Theme 3 Minority Status and Language and Theme 4 Housing Type and Transportation remained significant. Fourth, whether an individual will become ill and how severe their illness might be after eating *Salmonella*-contaminated ground beef can depend on the dose, characteristics of the *Salmonella* strain, and host factors, including underlying illness. Further, diagnosis of a *Salmonella* infection and inclusion in an outbreak requires access to and utilization of healthcare. People who live in rural areas might have less access to healthcare (MacKinney et al., 2014; Leider et al., 2020). Groups that have greater healthcare access may be overrepresented among outbreak-related cases, and people with more severe illness may be more likely to seek care. While we did not assess any of these factors in this analysis, they are important considerations in understanding who becomes sick and is ultimately included in ground beef-associated outbreaks. Lastly, we were unable to evaluate the types of retail locations (e.g., grocery stores, restaurants) that received contaminated ground beef and the demographic characteristics and social vulnerability of communities served by them, as retail locations that received contaminated ground beef are determined only for outbreaks in which a recall occurs.

## Conclusion

This report demonstrates how county-level SVI rankings can be useful to better understand the distribution of foodborne illness in the United States. County-level rankings for two SVI themes differed between patients and the general population, which suggests that stratifying by SVI theme is important to understand potential factors that could influence the risk for foodborne disease illness. Collecting and analyzing additional individual-level data and more complete and detailed demographic data, including further disaggregated race and ethnicity (e.g., tribal affiliation), could advance understanding of which social and demographic factors are most influential in foodborne illness. Applying SVI rankings to foodborne disease data also reinforces the importance of considering social determinants of health and drivers of health inequity, like language, household type, and transportation, in understanding potential risk factors for foodborne illness (CDC, 2024e). Interventions along the entire farm-to-fork continuum that reduce contamination of ground beef before it reaches consumers and consumer-focused interventions that are responsive to community and culture are needed to prevent foodborne illness.

## Supplementary Material

Appendix 1

## Figures and Tables

**Table 1:** Demographic characteristics of people infected with *Salmonella* linked to ground beef outbreaks—United States, 2012–2018

Outbreak no. (col%)											
Characteristic*	1	2	3	4	5	6	7	8	9	10	Total
**Year**	2012	2012	2012	2013	2014	2014	2017	2017	2018	2018	
**No. of illnesses**	51	22	24	39	3	47	107	5	3	436	737
**Sex**											
Male	28 (55)	10 (45)	9 (47)	19 (49)	3 (100)	19 (40)	53 (50)	2 (40)	0 (0)	222 (51)	365 (50)
Female	23 (45)	12 (55)	10 (53)	20 (51)	0 (0)	28 (60)	54 (50)	3 (60)	3 (100)	212 (49)	365 (50)
Unknown	0	0	5	0	0	0	0	0	0	2	7
**Median age (range)**	47 (3–101)	45 (2–87)	43 (8–68)	32 (<1–77)	9 (5–9)	34 (<1–85)	44 (<1–87)	37 (31–51)	64 (58–66)	42 (<1–99)	41 (<1–101)
**Age category (years)**											
<5	1 (2)	3 (14)	0 (0)	10 (26)	0 (0)	4 (9)	8 (7)	0 (0)	0 (0)	26 (6)	52 (7)
5–9	0 (0)	1 (5)	1 (5)	0 (0)	3 (100)	5 (11)	2 (2)	0 (0)	0 (0)	13 (3)	25 (3)
10–17	5 (10)	3 (14)	1 (5)	2 (5)	0 (0)	8 (17)	12 (11)	0 (0)	0 (0)	36 (8)	67 (9)
18–34	15 (29)	3 (14)	5 (26)	8 (21)	0 (0)	7 (15)	23 (21)	2 (40)	0 (0)	99 (23)	162 (22)
35–64	18 (35)	10 (45)	11 (58)	16 (41)	0 (0)	13 (28)	39 (36)	3 (60)	2	160 (37)	272 (37)
≥65	12 (24)	2 (10)	1 (5)	3 (8)	0 (0)	10 (21)	23 (21)	0 (0)	1	100 (23)	152 (21)
Unknown	0	0	5	0	0	0	0	0	0	2	7
**Race/ethnicity**											
Hispanic or Latino (any race)^†^	0 (0)	0 (0)	0 (0)	2 (6)	0 (0)	2 (6)	17 (19)	0 (0)	0 (0)	51 (14)	72 (12)
NH AI/AN	0 (0)	1 (9)	0 (0)	0 (0)	0 (0)	1 (3)	6 (7)	0 (0)	0 (0)	12 (3)	20 (3)
NH API	0 (0)	0 (0)	5 (100)	14 (39)	0 (0)	0 (0)	0 (0)	0 (0)	0 (0)	11 (3)	30 (5)
NH Black	4 (13)	1 (9)	0 (0)	0 (0)	0 (0)	0 (0)	2 (2)	5 (100)	0 (0)	8 (2)	20 (3)
NH multiple or another race(s)^‡^	0 (0)	0 (0)	0 (0)	0 (0)	0 (0)	1 (3)	1 (1)	0 (0)	0 (0)	6 (2)	8 (1)
NH White	27 (87)	9 (82)	0 (0)	20 (56)	2 (100)	31 (89)	63 (71)	0 (0)	3 (100)	274 (76)	429 (74)
Unknown	20	11	19	3	1	12	18	0	0	74	158
**Geographic classification** ^§^											
*Metropolitan*	32 (67)	12 (67)	0 (0)	25 (64)	3 (100)	35 (74)	88 (84)	5 (100)	3 (100)	373 (86)	576 (80)
Large central metropolitan	2 (4)	1 (6)	0 (0)	10 (26)	0 (0)	5 (11)	30 (29)	4 (80)	1 (33)	126 (29)	179 (25)
Large fringe metropolitan	9 (19)	5 (28)	0 (0)	4 (10)	1 (33)	12 (26)	14 (13)	1 (20)	2 (67)	83 (19)	131 (18)
Medium metropolitan	9 (19)	2 (11)	0 (0)	5 (13)	0 (0)	17 (36)	32 (30)	0 (0)	0 (0)	105 (24)	170 (24)
Small metropolitan	12 (25)	4 (22)	0 (0)	6 (15)	2 (67)	1 (2)	12 (11)	0 (0)	0 (0)	59 (14)	96 (13)
*Nonmetropolitan*	16 (33)	6 (33)	19 (100)	14 (36)	0 (0)	12 (26)	17 (16)	0 (0)	0 (0)	60 (14)	144 (20)
Micropolitan	9 (19)	1 (6)	0 (0)	6 (15)	0 (0)	7 (15)	12 (11)	0 (0)	0 (0)	28 (6)	63 (9)
Noncore/rural	7 (14)	5 (28)	19 (100)	8 (21)	0 (0)	5 (11)	5 (5)	0 (0)	0 (0)	32 (7)	81 (11)
*Unknown*	3	4	5	0	0	0	2	0	0	3	17

* Percentages calculated based on information available for each characteristic; percentages may not total 100 due to rounding. NH, non-Hispanic; AI/AN, American Indian or Alaska Native; API, Asian or Pacific Islander.

† Combined race/ethnicity was available for 69 patients who reported Hispanic or Latino ethnicity. Among these patients, 60 (87%) were White, 6 (9%) were multiple or another race(s), 1 (1%) was American Indian or Alaska Native, 1 (1%) was Asian or Pacific Islander, and 1 (1%) was Black. Among the six patients who reported Hispanic or Latino multiple or another race, five patients reported another unspecified race and one patient reported White and Black races.

‡ Among the eight patients who reported non-Hispanic multiple or another race, six patients reported another unspecified race, one patient reported American Indian or Alaska Native and Black races, and one patient reported Black and Asian races.

§ The 2013 National Center for Health Statistics Urban-Rural Classification Scheme for Counties was used to classify patients based on reported county of residence (Ingram & Franco, 2014).

**Table 2:** County social vulnerability index quartile among people infected with *Salmonella* linked to ground beef outbreaks—United States, 2012–2018*

Outbreak No. (col%)^†^											
CDC/ATSDR SVI quartile	1	2	3	4	5	6	7	8	9	10	Total No.
**Total cases per outbreak**	47	18	19	39	3	47	106	5	3	433	720
**Overall SVI**											
Q1 (lowest)	20 (43)	8 (44)	1 (5)	19 (49)	2 (67)	14 (30)	9 (8)	1 (20)	2 (66)	53 (12)	129 (18)
Q2	19 (40)	6 (33)	14 (74)	8 (21)	1 (33)	20 (43)	22 (21)	3 (60)	—	135 (31)	228 (32)
Q3	4 (9)	3 (17)	4 (21)	9 (23)	—	9 (19)	30 (28)	1 (20)	—	110 (25)	170 (24)
Q4 (highest)	4 (9)	1 (6)	—	3 (8)	—	4 (9)	45 (42)	—	1 (33)	135 (31)	193 (27)
**Theme 1 Socioeconomic Status**											
Q1 (lowest)	29 (62)	11 (61)	1 (5)	21 (54)	2 (67)	17 (36)	20 (19)	4 (80)	2 (66)	122 (28)	229 (32)
Q2	11 (23)	5 (28)	18 (95)	14 (36)	1 (33)	18 (38)	29 (27)	1 (20)	—	129 (30)	226 (31)
Q3	6 (13)	1 (6)	—	1 (3)	—	9 (19)	35 (33)	—	1 (33)	90 (21)	143 (20)
Q4 (highest)	1 (2)	1 (6)	—	3 (8)	—	3 (6)	22 (21)	—	—	92 (21)	122 (17)
**Theme 2 Household Composition and Disability**											
Q1 (lowest)	33 (70)	10	—	22 (56)	2 (67)	19 (4)	33 (31)	5 (100)	2 (66)	189 (44)	315 (44)
Q2	7 (15)	4 (22)	—	7 (18)	1 (33)	15 (32)	33 (31)	—	—	107 (25)	174 (24)
Q3	7 (15)	2 (11)	19 (100)	8 (21)	—	9 (19)	22 (21)	—	1 (33)	83 (19)	151 (21)
Q4 (highest)	—	2 (11)	—	2 (5)	—	4 (9)	18 (17)	—	—	54 (12)	80 (11)
**Theme 3 Minority Status and Language**											
Q1 (lowest)	12 (26)	6 (33)	—	7 (18)	—	9 (19)	4 (4)	—	—	7 (2)	45 (6)
Q2	8 (17)	3 (17)	1 (5)	12 (31)	1 (33)	8 (17)	10 (9)	—	2 (66)	30 (7)	75 (10)
Q3	16 (34)	5 (28)	14 (74)	7 (18)	1 (33)	15 (32)	10 (9)	1 (20)	—	67 (15)	136 (19)
Q4 (highest)	11 (23)	4 (22)	4 (21)	13 (33)	1 (33)	15 (32)	82 (77)	4 (80)	1 (33)	329 (76)	464 (64)
**Theme 4 Housing Type and Transportation**											
Q1 (lowest)	4 (9)	7 (39)	—	11 (28)	1 (33)	9 (19)	4 (4)	1 (20)	2 (66)	35 (8)	74 (10)
Q2	11 (23)	4 (22)	19 (100)	9 (23)	1 (33)	17 (36)	7 (7)	—	—	58 (13)	126 (18)
Q3	10 (21)	7 (39)	—	8 (21)	1 (33)	14 (30)	43 (41)	—	—	146 (34)	229 (32)
Q4 (highest)	22 (47)	—	—	11 (28)	—	7 (15)	52 (49)	4 (80)	1 (33)	194 (45)	291 (40)

* CDC/ATSDR SVI, Centers for Disease Control and Prevention/Agency for Toxic Substances and Disease Registry Social Vulnerability Index; —, no patients resided in counties within the SVI quartile. The CDC/ATSDR SVI ranks each county on 15 population-based social determinants of health measures and groups them into four themes: Theme 1: Socioeconomic Status, Theme 2: Household Composition and Disability, Theme 3: Minority Status and Language, and Theme 4: Housing Type and Transportation. Scores for SVI measures (overall and for each theme) represent percentile ranks by county ranging from 0 to 1, with higher scores indicating higher vulnerability. The analytic dataset excluded patients who were missing information on county of residence (*n* = 17). We attributed county-level SVI score to each individual patient by matching the patient illness onset date and county of residence to the SVI score from the most recent iteration. Scores for overall SVI and themes were analyzed as quartiles: first quartile (<25th percentile; lowest vulnerability), second quartile (25th–49th percentile), third quartile (50th–74th percentile), and fourth quartile (≥75^th^ percentile; highest vulnerability).

† Percentages calculated based on information available for each characteristic; percentages may not total 100 due to rounding.

**Table 3:** Weighted mean county social vulnerability index among people infected with *Salmonella* linked to ground beef outbreaks in the United States from 2012 to 2018 compared to the mean social vulnerability index among all US counties*

	Mean	95% CI	*P* value^†^
**Overall CDC/ATSDR SVI**	0.53	0.51–0.55	<0.001
Theme 1: Socioeconomic Status	0.42	0.40–0.44	0.99
Theme 2: Household Composition and Disability	0.35	0.33–0.37	0.99
Theme 3: Minority Status and Language	0.76	0.75–0.78	<0.001
Theme 4: Housing Type and Transportation	0.64	0.62–0.66	<0.001

* CDC/ATSDR SVI, Centers for Disease Control and Prevention/Agency for Toxic Substances and Disease Registry Social Vulnerability Index; CI, confidence interval. The CDC/ATSDR SVI ranks each county on 15 population-based social determinants of health measures and groups them into four themes: Theme 1: Socioeconomic Status, Theme 2: Household Composition and Disability, Theme 3: Minority Status and Language, and Theme 4: Housing Type and Transportation. Scores for SVI measures (overall and for each theme) represent percentile ranks by county ranging from 0 to 1, with higher scores indicating higher vulnerability. The analytic dataset excluded patients who were missing information on county of residence (*n* = 17). We weighted county-level SVI scores for counties with multiple patients by assigning each individual patient an SVI score that corresponded with their county of residence from the most recent iteration that matched their illness onset date. We calculated the mean and 95% confidence intervals (CIs) of the overall CDC/ATSDR SVI county-level rank and each theme among counties where ground beef outbreak-associated patients resided.

† Weighted mean SVI county-level rank among patients was compared to the mean SVI rank of all US counties (0.5; moderate vulnerability) using a one-sample inferiority *t*-test.

## Abbreviations:

ACS: American Community Survey
AI/AN: American Indian or Alaska Native
API: Asian or Pacific Islander
CDC/ATSDR SVI: Centers for Disease Control and Prevention/Agency for Toxic Substances and Disease Registry Social Vulnerability Index
CI: confidence interval
FDOSS: CDC Foodborne Disease Outbreak Surveillance System
NCHS: National Center for Health Statistics
NH: non-Hispanic
SEDRIC: System for Enteric Disease Response, Investigation, and Coordination.
