# Supplementary material for: Demographic Characteristics and County-level Indicators of Social Vulnerability in Salmonellosis Outbreaks Linked to Ground Beef—United States, 2012–2018
Source: J Food Prot. Author manuscript; Available in PMC 2025 Jan 28. (PMC11773426; doi:10.1016/j.jfp.2024.100411)
Supplement: Appendix 1 [file NIHMS2046432-supplement-Appendix_1.docx]

Appendix

Appendix Table 1. Demographic characteristics of people infected with *Salmonella* linked to ground beef outbreaks—United States, 2012–2018

| Characteristic* | Total  no. (%) | Total Excluding Outbreak 10  no. (%) |
| --- | --- | --- |
| **No. of illnesses** | 737 | 301 |
| **Sex** |  |  |
| Male | 365 (50) | 143 (48) |
| Female | 365 (50) | 153 (51) |
| Unknown | 7 | 5 |
| **Median age (range)** | 41  (<1–101) | 43  (<1–101) |
| **Age category (years)** |  |  |
| <5 | 52 (7) | 26 (8) |
| 5–9 | 25 (3) | 12 (4) |
| 10–17 | 67 (9) | 31 (10) |
| 18–34 | 162 (22) | 63 (21) |
| 35–64 | 272 (37) | 112 (38) |
| ≥65 | 152 (21) | 52 (18) |
| Unknown | 7 | 5 |
| **Race/ethnicity** |  |  |
| Hispanic or Latino (any race) | 72 (12) | 21 (10) |
| NH AI/AN | 20 (3) | 8 (4) |
| NH API | 30 (5) | 19 (9) |
| NH Black | 20 (3) | 12 (6) |
| NH multiple or another race(s) | 8 (1) | 2 (1) |
| NH White | 429 (74) | 155 (71) |
| Unknown | 158 | 84 |
| **Geographic classification**† |  |  |
| *Metropolitan* | 576 (80) | 203 (71) |
| Large central metropolitan | 179 (25) | 53 (18) |
| Large fringe metropolitan | 131 (18) | 48 (17) |
| Medium metropolitan | 170 (24) | 65 (23) |
| Small metropolitan | 96 (13) | 37 (13) |
| *Non-metropolitan* | 144 (20) | 84 (29) |
| Micropolitan | 63 (9) | 35 (12) |
| Noncore/rural | 81 (11) | 49 (17) |
| *Unknown* | 17 | 14 |

*Percentages calculated based on information available for each characteristic; percentages may not total 100 due to rounding. NH, non-Hispanic; AI/AN, American Indian or Alaska Native; API, Asian or Pacific Islander.

†The 2013 National Center for Health Statistics Urban-Rural Classification Scheme for Counties was used to classify patients based on reported county of residence [18].

Appendix Table 2. County social vulnerability index quartile among people infected with *Salmonella* linked to ground beef outbreaks—United States, 2012–2018*

| CDC/ATSDR SVI quartile | Total  no. (%)† | Total Excluding Outbreak 10  no. (%)† |
| --- | --- | --- |
| **Total cases per outbreak** | 720 | 287 |
| **Overall SVI** |  |  |
| Q1 (lowest) | 129 (18) | 76 (26) |
| Q2 | 228 (32) | 93 (32) |
| Q3 | 170 (24) | 60 (21) |
| Q4 (highest) | 193 (27) | 58 (20) |
| **Theme 1 Socioeconomic Status** |  |  |
| Q1 (lowest) | 229 (32) | 107 (37) |
| Q2 | 226 (31) | 97 (34) |
| Q3 | 143 (20) | 53 (18) |
| Q4 (highest) | 122 (17) | 30 (10) |
| **Theme 2 Household Composition and Disability** |  |  |
| Q1 (lowest) | 315 (44) | 126 (44) |
| Q2 | 174 (24) | 67 (23) |
| Q3 | 151 (21) | 68 (24) |
| Q4 (highest) | 80 (11) | 26 (9) |
| **Theme 3 Minority Status and Language** |  |  |
| Q1 (lowest) | 45 (6) | 38 (13) |
| Q2 | 75 (10) | 45 (16) |
| Q3 | 136 (19) | 69 (24) |
| Q4 (highest) | 464 (64) | 135 (47) |
| **Theme 4 Housing Type and Transportation** |  |  |
| Q1 (lowest) | 74 (10) | 39 (14) |
| Q2 | 126 (18) | 68 (24) |
| Q3 | 229 (32) | 83 (29) |
| Q4 (highest) | 291 (40) | 97 (34) |

*CDC/ATSDR SVI, Centers for Disease Control and Prevention/Agency for Toxic Substances and Disease Registry Social Vulnerability Index. The CDC/ATSDR SVI ranks each county on 15 population-based social determinants of health measures and groups them into four themes: Theme 1: Socioeconomic Status, Theme 2: Household Composition and Disability, Theme 3: Minority Status and Language, and Theme 4: Housing Type and Transportation. Scores for SVI measures (overall and for each theme) represent percentile ranks by county ranging from 0 to 1, with higher scores indicating higher vulnerability. The analytic dataset excluded patients that were missing information on county of residence. We attributed county-level SVI score to each individual patient by matching patient illness onset date and county of residence to the SVI score from the most recent iteration. Scores for overall SVI and themes were analyzed as quartiles: first quartile (<25^th^ percentile; lowest vulnerability), second quartile (25^th^–49^th^ percentile), third quartile (50^th^–74^th^ percentile), and fourth quartile (≥75^th^ percentile; highest vulnerability).

†Percentages calculated based on information available for each characteristic; percentages may not total 100 due to rounding.

Appendix Table 3. Weighted mean county social vulnerability index among people infected with *Salmonella* linked to ground beef outbreaks in the United States from 2012–2018 compared to the mean social vulnerability index among all US counties*

|  | Mean (95% CI) | p-value† | Mean (95% CI) Excluding Outbreak 10 | p-value† |
| --- | --- | --- | --- | --- |
| **Overall CDC/ATSDR SVI** | 0.53 (0.51-0.55) | <0.001 | 0.46 (0.44-0.48) | 0.985 |
| Theme 1: Socioeconomic Status | 0.42 (0.40–0.44) | 0.99 | 0.36 (0.34–0.38) | 0.999 |
| Theme 2: Household Composition and Disability | 0.35 (0.33–0.37) | 0.99 | 0.36 (0.33–0.39) | 0.999 |
| Theme 3: Minority Status and Language | 0.76 (0.75–0.78) | <0.001 | 0.66 (0.63–0.69) | <0.001 |
| Theme 4: Housing Type and Transportation | 0.64 (0.62–0.66) | <0.001 | 0.59 (0.56–0.62) | <0.001 |

*CDC/ATSDR SVI, Centers for Disease Control and Prevention/Agency for Toxic Substances and Disease Registry Social Vulnerability Index; CI, confidence interval. The CDC/ATSDR SVI ranks each county on 15 population-based social determinants of health measures and groups them into four themes: Theme 1: Socioeconomic Status, Theme 2: Household Composition and Disability, Theme 3: Minority Status and Language, and Theme 4: Housing Type and Transportation. Scores for SVI measures (overall and for each theme) represent percentile ranks by county ranging from 0 to 1, with higher scores indicating higher vulnerability. The analytic dataset excluded patients that were missing information on county of residence. We weighted county-level SVI scores for counties with multiple patients by assigning each individual patient an SVI score that corresponded with their county of residence from the most recent iteration that matched their illness onset date. We calculated the mean and 95% confidence intervals (CIs) of the overall CDC/ATSDR SVI county-level rank and each theme among counties where ground beef outbreak-associated patients resided.

†Weighted mean SVI county-level rank among patients were compared to the mean SVI rank of all US counties (0.5; moderate vulnerability) using a one-sample inferiority t-test.
